# An mHealth App and System Architecture for Respiratory Disease Management: Design Principles, Tool Development, and Pilot Usability Study

**DOI:** 10.2196/73584

**Published:** 2025-10-29

**Authors:** Andrew Chao, Lisa Martignetti, René Groh, Andreas M Kist, Nicole YK Li-Jessen

**Affiliations:** 1 School of Communication Sciences and Disorders Faculty of Medicine and Health Sciences McGill University Montreal, QC Canada; 2 Department of Biomedical Engineering McGill University Montreal, QC Canada; 3 Department of Artificial Intelligence in Biomedical Engineering Friedrich-Alexander-Universität Erlangen-Nürnberg Erlangen Germany; 4 Department of Otolaryngology—Head and Neck Surgery McGill University Montreal, QC Canada; 5 Translational Research in Respiratory Diseases Program Research Institute of McGill University Health Center Montreal, QC Canada; 6 The Centre for Research on Brain, Language and Music McGill University Montreal, QC Canada

**Keywords:** asthma, chronic obstructive pulmonary disease, COPD, development guidelines and standards, mobile health apps, usability assessment, wearable devices

## Abstract

**Background:**

Mobile health (mHealth) apps are software interfaces that enable users to access and manage wearable technology through smartphones and tablet devices for health improvement purposes. However, many respiratory disease mHealth apps lack transparent development documentation, compromising user confidence in their quality, functionality, and usability.

**Objective:**

This study aimed to develop and evaluate AIrway, a companion mHealth app designed to interface with an in-house wearable device for monitoring airway symptoms following established mHealth development and reporting standards.

**Methods:**

The development cycle of AIrway comprised 2 study phases. In phase 1, AIrway, a native Android app, was developed following academic and industrial standards (Android material design and Morville’s design principles) and privacy regulations (Personal Information Protection and Electronic Documents Act). Core functionalities included location-based environmental monitoring, a clinical diary with action plans, Bluetooth connectivity, and real-time data storage. In phase 2, the usability of AIrway was evaluated by software app developers using standardized assessment tools, namely, the User Version of the Mobile Application Rating Scale survey and the IQVIA questionnaire.

**Results:**

AIrway successfully fulfilled 7 of 8 development criteria on usability, privacy, security, appropriateness, transparency, safety, and technical support, with only the technology aspects requiring refinement. Accessibility assessments confirmed that AIrway’s content and interface were comprehensible to the general population (grade 9-10 reading level). Technical testing demonstrated reliable Bluetooth data transmission for up to 10 minutes without interruption. User evaluation scores for the User Version of the Mobile Application Rating Scale (3.6/5.0) and IQVIA (8/11) were comparable to those of similar mHealth apps on the market.

**Conclusions:**

By adhering to established mHealth app design principles, AIrway achieved the necessary accessibility standards and wireless communication capabilities for wearable device integration. Future development will focus on expanding cross-platform compatibility and conducting usability evaluation with intended patient populations to validate its clinical effectiveness and support ongoing improvements.

## Introduction

### Background

Asthma and chronic obstructive pulmonary disease (COPD) each affect approximately 330 million people worldwide, representing major public health challenges with substantial health and economic burdens [1,2]. Self-management interventions are key to mitigate the risk of symptom exacerbations in patients with asthma and COPD from escalating into life-threatening emergencies. To better support self-management, wearable technology can enable remote monitoring and early exacerbation detection, allowing patients to act before symptoms worsen [3-5].

Effective respiratory monitoring requires specialized sensing technologies that address unique challenges. While surface body sensors accurately capture vital signs including heart rate, skin temperature, and breathing patterns, continuous acoustic monitoring raises privacy concerns due to incidental speech recording [6-8]. Neck surface accelerometers (NSAs) provide an alternative approach, capturing purely mechano-acoustic signals without recording speech, making them suitable for long-term chronic disease monitoring [9-14]. Recent studies demonstrate that NSA-based systems can detect voice physiology, swallowing events, and COPD severity indicators with 93% to 96% accuracy using machine learning approaches [15-17].

The success of wearable systems depends on reliable mobile interfaces. In ubiquitous health solutions, raw sensor data (eg, from NSA) are stored onboard before transmission via Bluetooth Low Energy (BLE) to mobile devices for local processing or cloud-based analysis. Results are delivered through mobile health (mHealth) apps that often include interactive features such as self-assessment questionnaires and chatbots to promote healthier behaviors [18-21]. However, despite the increasing use of Android platforms for mHealth development, many studies lack comprehensive documentation of development workflows, design decisions, and validation procedures [22]. This transparency gap hinders clinical adoption, as mHealth apps serve as the primary interface between patients and wearable devices.

### Related Work and Research Gaps

Clinical research shows that wearable tools can increase medication adherence by 50% and improve disease-related knowledge by 52% [23]. However, Cochrane systematic reviews report mixed benefits for mHealth interventions targeting patients with asthma and COPD specifically.

Belisario et al [24] evaluated smartphone and tablet apps versus paper-based asthma action plans in 2 randomized controlled trials (n=408 participants) over 6 months. While emergency visits decreased significantly, symptom control showed no improvement on the patient-reported Asthma Control Questionnaire (ACQ). Likewise, mixed results are also noted in COPD research. Janjua et al [25] reviewed 14 randomized controlled trials (n=1518 participants) comparing mobile-based self-management to usual care. Evidence to date suggested that digital technology might provide short-term but not long-term improvements in patients’ quality of life. Nevertheless, these 2 Cochrane reports noted key methodological limitations that undermined the evidence quality in asthma and COPD literature, such as heavy reliance on self-reported outcomes, unregistered protocols, and underpowered statistics from small sample sizes [24,25].

Despite that many mHealth solutions are available, most of them remain inadequate to fully support self-management interventions in asthma and COPD [26]. General-purpose wearables such as the Apple Watch [27] and Fitbit [28] provide basic physiological metrics but lack the disease-specific algorithms and personalized interventions essential for respiratory disease management. Specialized devices such as Afflo [29] and AcuPebble [30] capture respiratory signals but do not integrate data into self-management plans or provide timely exacerbation alerts. Stand-alone apps like Breathe [31], Sonde Health [32], and CoughPro [33] rely on smartphone microphones and questionnaires for symptom detection. These methods capture only intermittent snapshots of respiratory status, rather than providing continuous monitoring capabilities.

More importantly, most mHealth solutions did not disclose how well their apps adhere to regulatory frameworks. Multiple standards govern mHealth apps, including the European Commission’s Green Paper on Mobile Health [34] and the US Food and Drug Administration’s Mobile Medical Applications Guidance [35], which establish privacy, encryption, and data-transfer requirements. Beyond regulatory compliance, developers shall follow established design principles found in industrial guidelines such as Apple’s Human Interface Guidelines [36] and Android’s Material Design Guidelines [37]. Besides, apps handling protected health information must comply with the Health Insurance Portability and Accountability Act (HIPAA) or equivalent regional privacy regulations [38]. To address these multiple requirements, Llorens-Vernet and Miró [39] developed a comprehensive framework that consolidates these diverse requirements into 8 unified development criteria: usability, privacy, security, appropriateness and suitability, transparency and content, safety, technical support and updates, and technology.

The aforesaid research gaps, namely, suboptimal clinical evidence, technical constraints, and inadequate development documentation, underscored the need for more rigorously developed mHealth solutions in respiratory disease monitoring. Our team previously developed privacy-preserving NSA-enabled wearable sensors for airway symptom detection [12,13,25,40]. However, these sensors require a companion mobile app to enable data visualization, cloud storage, user interaction, and so on. This study aimed to address these technological gaps by developing and evaluating a mobile app using transparent, framework-guided development processes.

### Research Objectives

This study presented the development and evaluation of AIrway, an mHealth app for asthma and COPD management. Research objectives were (1) to develop the app that would collect patient-reported outcomes, environmental data, and peripheral accelerometer sensor data; (2) to evaluate the functionality of the app including wireless connectivity, cloud storage, and literacy analysis; and (3) to perform a pilot usability study with technical raters, namely, app developers.

## Methods

### Study Design

This study consisted of 2 phases, namely, frontend and backend development (phase 1) and a pilot usability test (phase 2). The AIrway development followed rigorous academic and industry best practices to ensure compliance with the mHealth development standard. Comprehensive documentation of app requirements, design, implementation and validation, and benchmark were reported against the framework of Llorens-Vernet and Miró [39].

### Phase 1a: Frontend Development

#### App Interface Implementation and Workflow

**Figure 1:**
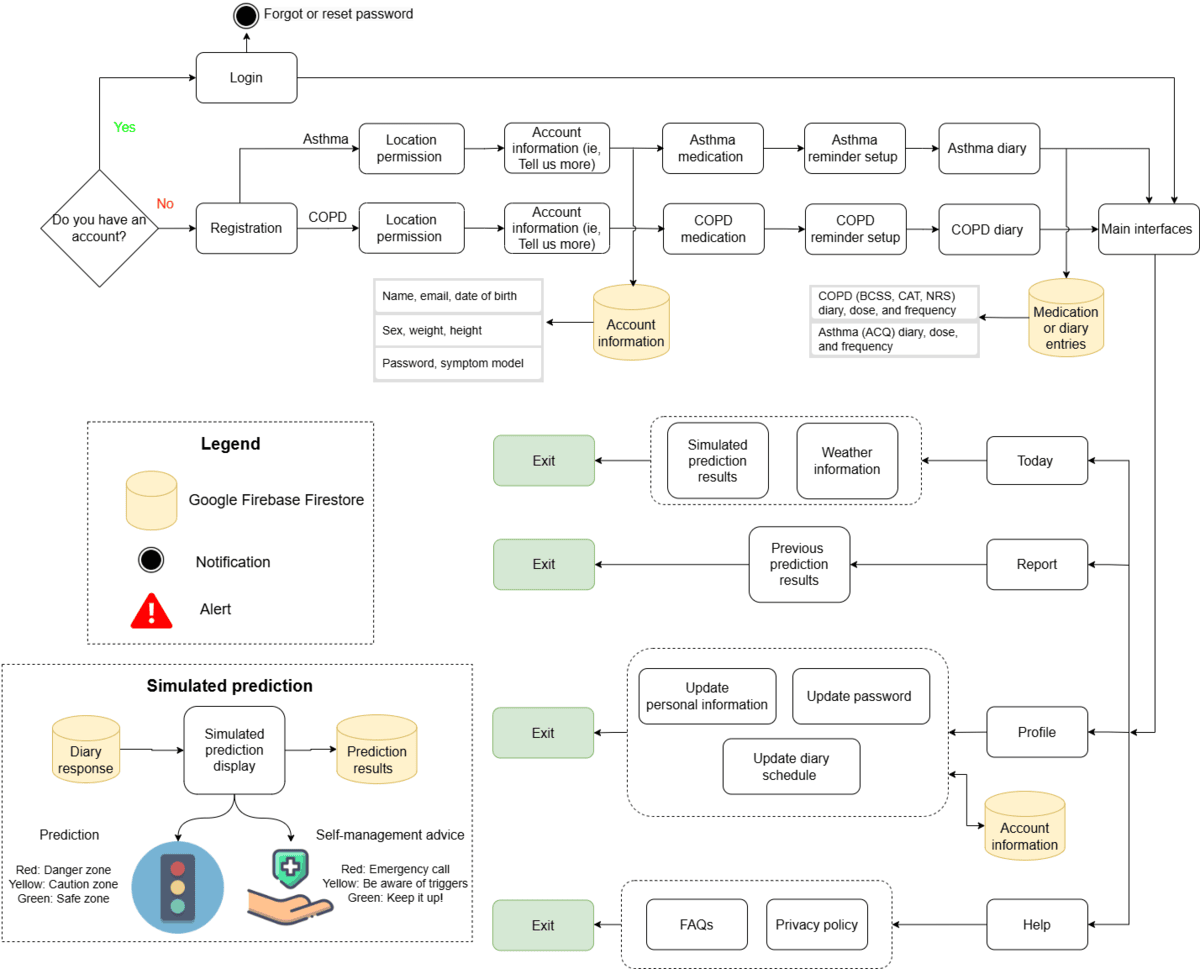
The design flowchart of the AIrway app. The login and registration interface allowed users to input their login information and create an account. The location permission interface allowed users to turn on or off the device’s location for parsing local weather information. The account interface allowed users to complete the profile setup. The app also had interfaces today, report, profile, and help for data visualization and information delivery. ACQ: Asthma Control Questionnaire; BCSS: Breathlessness, Cough and Sputum Scale; CAT: COPD Assessment Test; COPD: chronic obstructive pulmonary disease; FAQ: frequently asked question; NRS: Numeric Rating Scale.

The AIrway user interface (UI) was set up using XML (version 1.0) [41] in Android Studio (Chipmunk 2021.2.1 version) [42], a Google native Android development integrated development platform. The integrated development platform allowed developers to build Android apps on Windows, Mac, and Linux operating systems. The app supported respiratory disease management with 2 types of interfaces. Task-based interfaces allowed users to create a personalized account and log in, manage location permissions, input medication details, log symptoms via a clinical diary, and set diary reminders. Meanwhile, visualization interfaces displayed local weather, action plans, and health data summaries and provided account and app privacy information (Figure 1).

#### Design Principles

The design principles of Morville [43] were followed to guide the user experience (UX) and UI design of AIrway (Table 1). In brief, Morville’s principles include 7 aspects of UX for mobile apps and websites: usefulness, value, findability, credibility, accessibility, desirability, and usability. These principles have been widely adopted in mHealth apps such as an emotional and physical health support app for older people [44] and a posttraumatic stress monitoring app [45].

**Table 1:** Design principles of the AIrway appa.

Design principle	Question (Q) and answer
Usefulness	Q1: Does the app have practical value for the target users? The app allowed the target users (ie, patients with asthma or COPDb) to create a personalized account, monitor their conditions, and predict airway conditions through a clinical diary. It also provided reminders and alerts for diary completion. The app stored the data in a cloud service.
Value	Q2: Does the app advance the mission of the organization behind it? The AIrway app was developed by the Voice and Upper Airway Research Lab at McGill University, Canada, in collaboration with researchers at the University of Erlangen-Nürnberg, Germany. We are improving personalized medicine in voice and upper airway dysfunctions with advanced technology. For this project, we hope to support asthma and COPD management and provide recommended actions to the user.
Findability	Q3: Can users locate what they are looking for? The app was developed using large fonts, buttons, and a navigation menu to assist the user to switch between interfaces. The app also incorporated some frequently asked questions to support the user.
Credibility	Q4: Is the app trustworthy? The app components were developed based on other mHealthc apps, such as Fitbit [28], Afflo [29], and Breathe [31]. The clinical diary and prediction results were developed based on clinical guidelines and literature (such as GINAd and GOLDe). Data are safely stored in Google Cloud Services, which are eligible for HIPAAf compliance.
Accessibility	Q5: Are there barriers that may prevent the target users from using the app? The app was developed based on the WCAGg [46] and readability metrics. For instance, our app used user-friendly colors for the app background and easily understandable written materials for the app content.
Desirability	Q6: Do the target users want to use the app? What are the responses? A usability study was conducted with app developers. The study protocol was outlined, and the responses were summarized in the Results section.

^a^A question and answer format was presented to summarize the adaption of Morville’s design principles to the app’s user experience and user interface elements in this study [43].

^b^COPD: chronic obstructive pulmonary disease.

^c^mHealth: mobile health.

^d^GINA: Global Initiative for Asthma.

^e^GOLD: Global Initiative for Chronic Obstructive Lung Disease.

^f^HIPAA: Health Insurance Portability and Accountability Act.

^g^WCAG: Web Content Accessibility Guidelines.

For AIrway, the end users are individuals with asthma or COPD, who are often older people. To apply Morville’s principles for the app, the UX and UI were built with simplified UIs, large texts, easy-to-use navigation buttons, and motivational functions, such as reminders to keep users engaged and encouraged for continued use [47].

#### Optimal Element Layout, Text Font, and Color Contrast for App Interfaces

The Android operating system was chosen for deploying AIrway across different devices, such as LG, Google Pixel, and Samsung. As these devices can vary significantly in their screen size, the app content display can be altered, and information may become misaligned.

To address this development issue, the app’s UI was built using the constraint layout [48] following the Android Material Design Guidelines [37]. Constraint layout allows UI elements (eg, buttons and texts) to be positioned relative to each other. For instance, the elements can always be specified relative to the top, center, or bottom of the screen using constraint layout in the XML (version 1.0) file [48]. That way, the app’s UI components were flexible enough to be adjusted and fit different screen sizes. Besides, Android Guidelines [37] recommend a minimum text size of 20 scalable pixels for titles, 16 scalable pixels for subtitles, and 14 scalable pixels for buttons and body to improve readability. These recommendations were adhered to in the development of the app.

Color contrast is critical for user accessibility and usability. According to Web Content Accessibility Guidelines [46], texts and images should have a minimum contrast ratio of 7:1 (ie, AAA compliance). The app’s UI components, such as backgrounds, buttons, drop-down menus, and input fields, were adjusted using an open-source color contrast checker [49] to meet or exceed this requirement. All interface elements achieved contrast ratios between 12:1 and 21:1, ensuring high visibility for users with low vision or reduced contrast sensitivity.

#### User Login and Registration

Upon using the app, users first interacted with the login interface. They could either log in with their credentials or create an account on the register interface by entering their name, email, and password and selecting a condition to monitor. The email input field included validation checks for proper formatting, and passwords were masked for security. Based on their selection, users were directed to either asthma or COPD-specific interfaces. If users forgot their password, they could request a reset link via a valid email and then log in with the new password (Multimedia Appendix 1).

#### Location Permission

After registration, the app requested access to the device’s location to retrieve local weather data. This location permission was declared in the AndroidManifest.xml file, with ACCESS_COARSE_LOCATION enabling access to the approximate location without storing it [50]. The app requested the permission at runtime, offering 3 options: “While using the app,” “Only this time,” or “Deny” (Multimedia Appendix 1).

#### User Account Information

Users provided personal information including their biological sex, date of birth, weight, and height. Biological sex was a drop-down menu with 3 options: “Male,” “Female,” and “Prefer not to say.” A DatePicker [51] component allowed users to easily scroll through dates for their date of birth. Input fields for weight and height accepted only numbers, with selectable units: “kg” or “lbs” for weight and “m” or “ft” for height (Multimedia Appendix 1).

#### Medication Profile

The medication profile design was based on the guidelines from the Canadian Lung Association [52], the Ontario Lung Association [53], and the Breathe app [31]. Depending on the condition, users filled out either the asthma or COPD profile including the medication name, amount, and frequency. To handle over 10 options without cluttering the UI, a drop-down menu was implemented. This design allowed users to easily select a suitable option with a single click, after which the menu collapsed to display only the selected option, providing a clean UI to users (Multimedia Appendix 1).

#### Clinical Diary

Effective self-management and improved health care outcomes for asthma and COPD rely on clinically validated diaries that are user-friendly, relevant to the patient’s symptoms and medications, and capable of identifying the predictors of exacerbations [54,55]. The app prompted users to fill out the clinical diary (asthma or COPD) daily either after the initial registration or at the scheduled time.

For asthma, it used the ACQ [56], a clinically validated tool recommended by the Global Initiative for Asthma (GINA) [57], comprising 5 items for assessing asthma symptoms and 1 item for rescue inhaler bronchodilator use. For COPD, it incorporated 3 questionnaires: the COPD Assessment Test [58], the Breathlessness, Cough and Sputum Scale [59], and a Numeric Rating Scale [60] for a total of 12 items.

**Figure 2:**
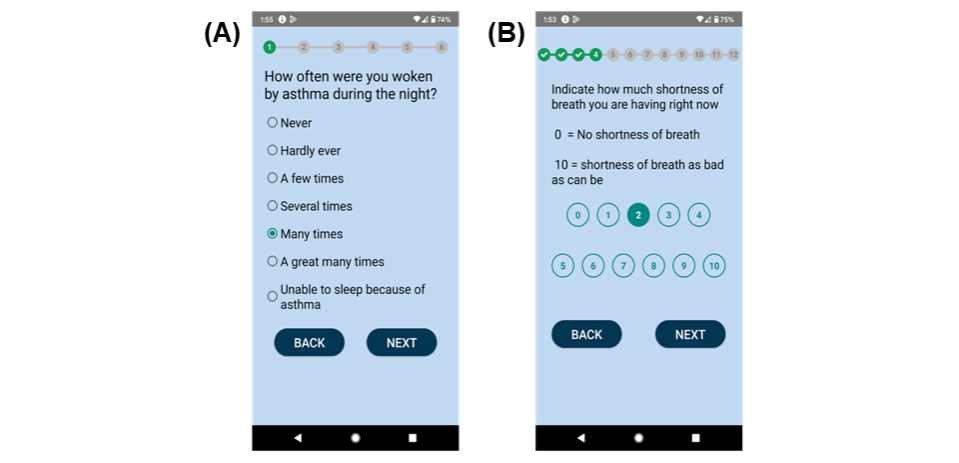
Clinical diary interfaces of AIrway. (A) An Asthma Control Questionnaire question is presented in the asthma diary interface. (B) The Numerical Rating Scale question is presented in the chronic obstructive pulmonary disease diary interface. The diary interface presented 1 question per page with “Back” and “Next” buttons along with a progress bar.

The diary interface presented 1 question per page with “Back” and “Next” buttons along with a progress bar. Responses used RadioButton or Likert-scale styles for single-choice responses, in which users could change or deselect answers. If users tried to move on without answering, a pop-up message “Please select one choice” would appear (Figure 2). A TimePicker [61] component allowed users to schedule a daily reminder. At the scheduled time, a notification would appear with the message “Please complete the diary now!” offering 2 buttons: “Complete” (to open the diary) and “Dismiss” (Multimedia Appendix 1).

#### Today Interface: Local Weather Information and Control Zone Results

**Figure 3:**
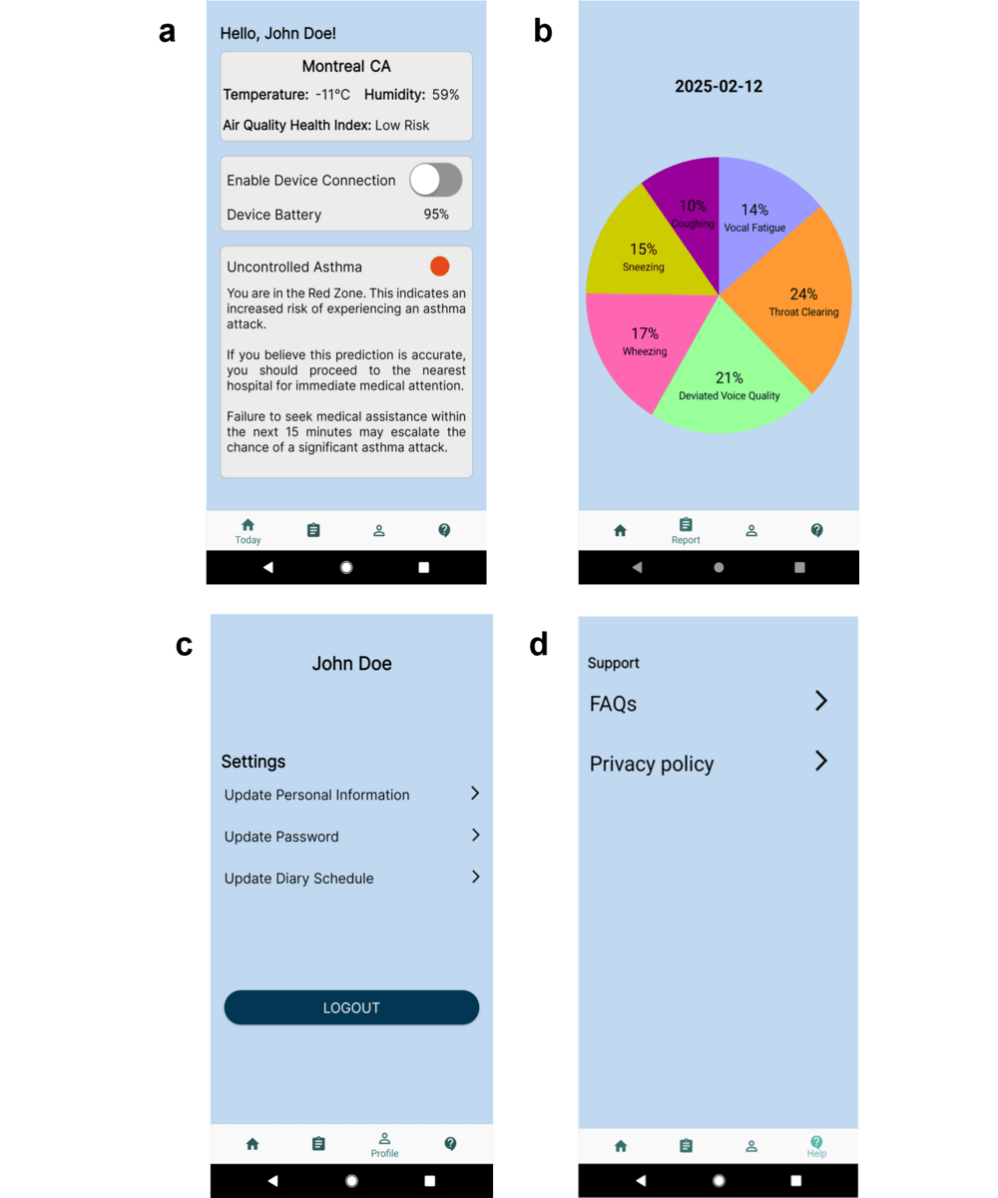
AIrway visualization interfaces. (A) Today, (B) report, (C) profile, and (D) help interfaces were designed for information delivery.

The today interface featured both local weather information and the asthma or COPD control zone of the day (Figure 3A). Since asthma or COPD symptoms can be impacted by air pollution, temperature, and humidity [62], the interface incorporated key air quality indicators outlined by the World Health Organization guidelines, including particulate matter (PM), ozone (O_3_), sulfur dioxide (SO_2_), and nitrogen dioxide (NO_2_) [63]. In Canada, Environment Canada’s Air Quality Health Index (AQHI) is used to convey the health impact of air quality. This AQHI information was retrieved and displayed on the today interface [64].

#### Report Interface: Clinical Symptom Prediction

The report interface (Figure 3B) featured a pie chart representing the percentages of each symptom. These numbers, for instance, could represent symptom data sourced from an external wearable device or body sensor via wireless BLE communication technology.

#### Profile Interface: Account Information Management

The profile interface (Figure 3C) allowed users to view the information they entered during the previous profile setup by clicking the corresponding texts or arrow icons. In addition, users could manage their accounts and update the diary schedule as well as log out of their accounts by clicking the “Logout” button.

#### Help Interface: Support, Resources, and Data Privacy

The help interface (Figure 3D) provided users with detailed information on the app’s privacy policy and frequently asked questions (FAQs). The privacy policy, developed in line with the Personal Information Protection and Electronic Documents Act [65] and Quebec Privacy Law [66], explained the type of data collected as well as how it would be stored and used (Multimedia Appendix 2). The FAQs provided additional explanations on the development of asthma and COPD zone calculations and technical support (eg, reset password procedure). It also included the research team’s contact email for additional inquiries.

In addition, the Google Firebase Firestore database [67] used for app data storage is HIPAA [68] compliant. Google will strictly adhere to the US national standards to protect health information and data through the Business Associate Agreement. The privacy policy also included the Business Associate Agreement information.

#### Literacy Analysis for the App’s Written Materials

The app’s written materials, including the privacy policy, FAQs, the asthma action plan, and the COPD action plan, were evaluated using an open-source readability calculator, Online-Utility [69]. The target reading level was set to grade 9, matching most English-educated adults in the United States [70]. The Flesch Reading Ease Score, a standard metric for research, educational, and digital content [71], was applied (70-80=grade 7, 60-70=grades 8-9, and 50-60=grades 10-12) [72]. All text was iteratively revised until it consistently met the ninth-grade threshold without losing essential detail. Definitions for each readability metric appear in Multimedia Appendix 3.

#### Mobile Accessibility Analysis for App Interfaces

Based on the World Wide Web Consortium guideline [73,74], the content of websites and apps needs to be accessible to people with disabilities and the general population. The Accessibility Scanner [75], the official Android accessibility testing tool available on the Google Play Store, was used to verify the accessibility of the app. The scanner took a snapshot of the target interface and evaluated its components, such as content labels, target size, clickable items, and text and image contrast. The scanner then provided content-based descriptions and recommendations for improvement. For example, one suggestion was to adjust the item’s height from 32 to 48 density-independent pixels or larger.

### Phase 1b: Backend Development

The backend development was programmed using Java (version 11.0.12) [76] in Android Studio (Chipmunk 2021.2.1 version) [42] to implement (1) local weather data retrieval, (2) asthma and COPD control zone calculations, (3) cloud database storage and retrieval, and (4) BLE data transmission.

#### Local Weather Data Retrieval

The app retrieved the current local weather information (temperature and humidity) and used the current PM_2.5_, O_3_, and NO_2_ to compute the AQHI. The information was displayed on the today interface using the OpenWeatherMap application programming interface (One Call Version 3.0), an online service that provides the latest forecast data and air pollution information in over 200,000 cities for web and mobile app developers [77].

#### Control Zone Calculations and Generation of Action Plans

For asthma, the inputs for control zones (red, yellow, and green) included (1) user responses from the ACQ [56], (2) air quality data from the AQHI [64], as well as (3) the temperature and humidity levels of user’s current city location. The ACQ provides a mean score determining asthma severity, while the AQHI indicates health risk levels, with high readings exacerbating symptoms [56,78]. Extreme temperature and humidity can also trigger asthma symptoms [79]. Additionally, the app categorized asthma severity into “Uncontrolled,” “Partly controlled,” and “Well controlled” based on GINA guidelines, providing corresponding self-management action items from the Centers for Disease Control and Prevention asthma action plan [80] (Multimedia Appendix 4).

For COPD, the inputs for control zones (red, yellow, and green) included (1) user responses from the 3 validated questionnaires: the Breathlessness, Cough and Sputum Scale [60], the COPD Assessment Test [58], and the Numeric Rating Scale [81]; (2) air quality data from the AQHI; as well as (3) the temperature and humidity levels of user’s current city location. These questionnaires assess the impact of COPD symptoms, which can be worsened by extreme temperature and humidity. Self-management action items for each control zone were provided based on the Canadian Thoracic Society COPD action plan [82] (Multimedia Appendix 4).

#### Cloud Database

mHealth apps often integrate cloud databases for data storage, processing, and sharing to ensure ease of access and scalability. In the AIrway app, Google Firebase Firestore [67] was used to store large volumes of real-time user data, such as account information and clinical diary responses. Firestore was considered an ideal cloud database for the app for 3 reasons.

Firestore was chosen for 3 main reasons. First, it provides real-time updates and integrates directly into common mobile development frameworks. Researchers have also used Firestore in an eHealth management system for cardiovascular diseases [83] and a humidity and temperature monitoring system [84]. Second, its Not Only Structured Query Language design requires no fixed schema: collections and documents can grow independently and hold diverse data types, from text and numbers to arrays and objects [85-87]. Third, unlike traditional Structured Query Language databases that need hardware upgrades and incur downtime, Firestore scales horizontally on demand, adding capacity without service interruption [88].

#### Cloud Database Access Feature

To access Firestore, users must authenticate via the Firebase Authentication Service using credentials such as email and password, phone number, and third-party providers (eg, Google and Facebook) [89]. In AIrway, the register interface collected email and password inputs. Once the user input fields were filled, the createUserWithEmailAndPassword method [89] was used to create an account. Upon successful registration, the message “User successfully created!” was displayed, while a failure showed “This account already exists. Please register another email!” preventing users from moving to the next interface. The registered email and password then served as the user’s sign-in key for logging in and out of Firestore.

#### Cloud Data Storage and Data Retrieval

**Figure 4:**
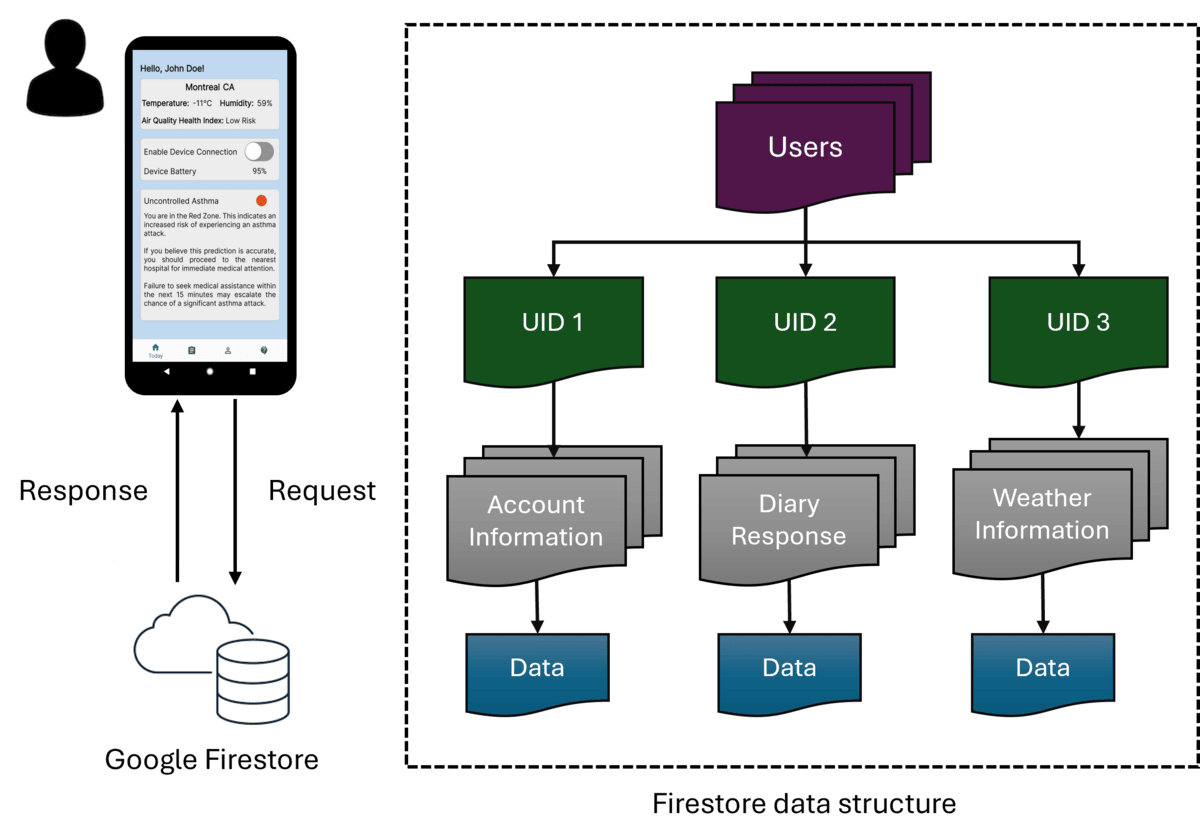
Firestore cloud architecture and data flow of the AIrway app. The storage structure comprised a “users” collection containing individual documents identified by authentication UIDs. UID: user ID.

Storing app data in Firestore involved 3 steps. First, read or write access was enabled by setting the rules to “True” for authenticated users. Second, in Android Studio, a storage path was defined with a collection named “users” and a document named after the user ID generated during authentication. User data were written within this collection, with each user’s specific data stored in a separate document (Figure 4). Third, new data objects were added using the Map <String, Object> structure and push methods. For example, an asthma diary question like “How bad were your asthma symptoms when you woke up in the morning?” was stored with the selected RadioButton response under the header “Asthma Diary Response.”

The app also retrieved user data, such as the account details in the profile interface, to enhance data visualization and increase user retention. This feature allowed users to quickly access personalized data and review previous entries before making updates. For example, when updating a password, the app reminded users of their current password to prevent them from setting the same one again. To achieve this dynamic data retrieval feature, the app first checked if the user document existed. If it did, the getString method fetched the corresponding data in the app. The setHint method then displayed the stored data.

#### BLE Communication Features and Testing

BLE data transmission enables the app to receive data from an external wearable device, that is, NSA in this study with minimal power use [90,91]. The BLE protocol stack [92] has 2 sections. The controller is at the lower level of the stack that handles radio transmission and manages the data connection between 2 devices, while the physical layer is responsible for analog communication operations [93]. The host operates at a higher level of the stack that defines the data transmission between a device (peripheral) and a mobile app (client) using the Attribute Protocol and the Generic Attribute Profile. The Attribute Protocol allows for data request between the client and the peripheral, whereas the Generic Attribute Profile organizes data into services and characteristics [91,94,95].

In the AIrway app, the Android BLE application programming interface [96] handled scanning, connecting, and reading functions. Users clicked a button to scan for nearby devices, and the app listed available devices, showing each device’s name, Media Access Control address (ie, a hardware identifier that uniquely identifies each device), and its Received Signal Strength Indicator measured in decibel-milliwatts (dBm). After selecting a target device, the app established a connection using the connectGatt method [97], read the characteristics (ie, numerical data) using the readCharacteristic method [98], and displayed the characteristic values in real time. To optimize storage, only the first bit of each 16-bit characteristic representing the actual value was stored in Firestore.

For testing, a prototype printed circuit board based on the nRF52840 development board [99] was used to simulate 2 services, namely, symptom event classification (sending random integers every half second) and battery level (0 to 100). This setup verified reliable BLE data transmission to the app.

### Phase 2: Functionality Usability Tests With Technical Raters

A pilot usability study with technical raters (app developers) was performed to evaluate the app’s pilot functionality.

#### Implementation of App Usability Survey

The User Version of the Mobile Application Rating Scale (uMARS) [100] and IQVIA [101] were used. The uMARS questionnaire, widely used for evaluating mHealth apps [100], is structured into 5 sections: engagement, functionality, aesthetics, information quality, and subjective quality. In this study, the original uMARS questionnaire was modified for clarity and to ensure its relevance to the specific functions of the app with an open-text prompt for feedback (Multimedia Appendix 5). In addition, the IQVIA questionnaire was used to assess the presence of specific app functions, with a particular focus on self-management features such as alerts, guidance, and communication [101] (Multimedia Appendix 6).

The usability survey was deployed using McGill’s IT-managed LimeSurvey platform (version 3; LimeSurvey GmbH) and contained 51 items in total. The survey included prescreening (5 items), consent (1 item), and 4 separate sections. Sections A and B measured technical raters’ demographics (6 items) and mobile app development experience (5 items). Section C included the uMARS questionnaire (23 items), while Section D included the IQVIA questionnaire (11 items).

#### Study Recruitment and Procedure

A convenient sample of app developers was recruited via an electronic advertisement that was disseminated through Canadian universities’ computer science student mailing lists and social media. Inclusion criteria were (1) being aged 18 years and older; (2) at least 1 year of mobile app development experience or, for computer science PhD students and postdoctoral fellows, completion of a mobile app development course or related thesis research; (3) Android smartphone user with internet access; and (4) use of English as the primary daily language. Exclusion criteria included severe cognitive and psychiatric conditions that may prevent study completion. Technical raters were compensated with a US $35 Amazon gift card.

During the 1.5-hour Zoom (Zoom Video Communications) session, a study investigator (AC) explained the workflow. The raters received a fictitious Gmail address to access an instruction manual. After completing prescreening, consent, demographics, and mobile app development sections in LimeSurvey, they received an email from Firebase App Distribution [102] to download the app. Once the tasks were completed, they filled out the uMARS and IQVIA questionnaires in LimeSurvey.

### Ethical Considerations

The human study was approved by the institutional review board of the Faculty of Medicine and Health Sciences at McGill University (protocol A12-E39-22B). Informed consent was obtained from all participants. Participant data were deidentified and anonymized for privacy protection. Each received a US $35 Amazon gift card as compensation.

## Results

### Verification of mHealth Apps’ Development Standards

AIrway met 7 of the 8 best practices for mHealth app development (Table 2). Usability compliance was confirmed through a technical-expert evaluation and strict adherence to Android design guidelines. As the app targeted English-speaking adults with respiratory conditions, multilingual support was not included.

**Table 2:** Comparative results of AIrway app against established mobile health development standards [39].

		AIrway
**Usability criterion**		
	The app has been tested by potential users before being made available to the public.	Yes (usability study)
	It has instructions or some kind of assistance for use.	Yes (Help page)
	It is easy to use (ie, navigation is intuitive).	Yes (usability study)
	It follows the recommendations, patterns, and directives in the official manuals of the different operating systems (Android, iOS, or others).	Yes (Android development guidelines)
	The interface design follows the same pattern. That is, all graphic elements (typographies, icons, and buttons) have a consistent appearance. The function of each element (navigation menu, lists, and photo gallery) is clearly identified.	Yes
	The functionality is adapted to the purpose of the app.	Yes
	The information of the app must be able to be accessed in the shortest possible time. All users must be able to access all resources regardless of their capabilities.	Yes
	The app can be consulted in more than 1 language. All languages adapt appropriately to the content interface.	Not applicable
**Privacy criterion**		
	The app gives information about the terms and conditions of purchases in the app and the personal data recorded.	Yes
	It gives information about the kind of user data to be collected and the reason (the app must only ask for user data that are essential for the app to operate). It gives information about access policies and data treatment and ensures the right of access to recorded information. It describes the maintenance policy and the data erasure procedure. It gives information about possible commercial agreements with third parties.	Yes (consent form or privacy policy)
	It guarantees the privacy of the information recorded. It requires users to give their express consent. It warns of the risks of using the app.	Yes (privacy policy)
	It tells users when it accesses other resources of the device, such as their accounts or their social network profile.	Not applicable
	It takes measures to protect minors in accordance with the current legislation.	Not applicable
	Confidential user data are protected and anonymized, and there is a privacy mechanism so that users can control their data.	Yes (anonymized ID or fictitious names)
**Security criterion**		
	The app has encryption mechanisms for storing, collecting, and exchanging information. It has password management mechanisms.	Yes (password protection in the cloud)
	The cloud services used have the relevant security measures. It states the terms and conditions of cloud services.	Yes (Firestore cloud terms or conditions)
	The authorization and authentication mechanisms protect the users’ credentials and give access to their data. It limits access to data that are only necessary for the user.	Yes (Firestore account credentials or login)
	It detects and identifies cybersecurity vulnerabilities, possible threats, and the risk of being exploited. It applies the appropriate security measures to cybersecurity vulnerabilities in the face of possible threats.	Not applicable
**Appropriateness and suitability criteria**		
	The end users for whom the app is designed are explicitly indicated or actually intuitable (the name identifies the app) to the audience to whom it is set out.	Yes
	The benefits and advantages of using the app are explained.	Yes
	The app has been validated or created by experts (eg, a group of specialized professionals, a health organization, or a scientific society).	Partially yes (usability study with developers)
**Transparency and content criteria**		
	The app identifies the authors of the content and their professional qualifications.	Yes (Help page or FAQ^a^)
	It gives transparent information about the owners’ identity and location.	Yes (Help page or FAQ)
	It gives information about its sources of funding, promotion, and sponsorship, and possible conflicts of interest. Any third parties or organizations that have contributed to the app development are clearly identified.	Yes (Help page or FAQ)
	It uses scientific evidence to guarantee the quality of the content. It is based on ethical principles and values.	Yes
	The sources of the information are indicated. Concise information is given about the procedure used to select the content.	Yes
**Safety criterion**		
	The possible risks to users are identified. Users are warned that the app does not intend to replace the services provided by a professional.	Yes (action plan)
	Potential risks for users caused by bad use or possible adverse effects are explained.	Yes
**Technical support and updates criteria**		
	It gives a warning if updates modify or affect how the app functions. It gives a warning if updates can influence insensitive data.	Yes (Firebase App Distribution can send updates)
	Frequent security updates are guaranteed. Every time an update of a third-party component is published, the change is inspected, and the risk evaluated.	Yes (Firebase App Distribution can send updates)
	The frequency with which the content of the app is revised or updated.	Yes (Firebase App Distribution can send updates)
	Users have support mechanisms (email, phone, and contact form) for solving doubts, problems, or issues related to the health content and technical support.	Yes
**Technology criterion**		
	It works correctly. It does not fail during use (eg, blocks). Functions are correctly retrieved after context changes (eg, switch to another app and return), external interruptions (eg, incoming calls or messages), and switching off the terminal.	Yes
	It does not waste resources excessively: battery, central processing unit, memory, data, or network.	Partially yes (battery use with BLE^b^, memory use)
	It can work in flight mode and deal with network delays and any loss of connection.	No (need internet to log in and connect to the cloud)
	It supports multiple versions of data structures.	Yes (graph, text, and button)
	It supports multiple formats (eg, to support different operating systems).	No (Android only)

^a^FAQ: frequently asked question.

^b^BLE: Bluetooth Low Energy.

Privacy is protected by a comprehensive policy that covers data collection, terms of use, and secure cloud storage. Security relies on password-protected Google Firestore access, although broader cyberthreat defenses remain to be implemented. The app’s appropriateness for English-speaking adults with respiratory conditions was demonstrated via targeted performance testing.

Transparency requirements were satisfied through dedicated Help and FAQ sections providing author credentials, mission statements, funding information, and scientific evidence supporting the asthma and COPD control zone calculations. Safety protocols included explicit disclaimers regarding clinical diagnosis limitations and emergency response guidance for users experiencing severe symptoms.

Technical support is provided via monitored email channels and app updates distributed through Firebase App Distribution. The only area needing enhancement is technology—specifically, adding cross-platform compatibility and offline functionality, since AIrway is currently Android-only and requires a constant internet connection.

### Verification of Literacy Analysis

Based on the readability calculator [69], the Flesch Reading Ease score [103] for the privacy policy, FAQs, the asthma action plan, and the COPD action plan were 58.63, 56.69, 57.72, and 59.91, respectively. With an average score near 60 (1.4), these materials are accessible to users with a formal education level of grades 9-10. The Gunning Fog Index [104] indicated an 8th- to 10th-grade reading level, while the Coleman Liau Index [105] suggested suitability for a 7th- to 10th-grade audience. The Flesch-Kincaid Grade Level [106] indicated a 6th- to 8th-grade range, and the Automated Readability Index [72] estimated a 5th- to 7th-grade level. The Simple Measure of Gobbledygook Index measured the texts based on syllables [107] and concluded that the materials were suitable for a 9th- to 10th-grade audience (Table 3).

**Table 3:** Results of literacy analysis on AIrway app materials based on 5 standard readability indices [108].

Readability metrics	Privacy policy	FAQ^a^	Asthma action plan	COPD^b^ action plan	Interpretation of the metric
Gunning Fog Index	8.97	9.38	8.44	8.49	8th to 10th grade
Coleman Liau Index	9.18	9.39	7.47	8.35	7th to 10th grade
Flesch-Kincaid Grade Level	7.57	8.03	7.17	6.80	6th to 8th grade
Automated Readability Index	5.94	6.33	4.86	5.38	5th to 7th grade
SMOG^c^ Index	9.32	9.67	9.06	9.71	9th to 10th grade

^a^FAQ: frequently asked question.

^b^COPD: chronic obstructive pulmonary disease.

^c^SMOG: Simple Measure of Gobbledygook.

### Accessibility Results

The accessibility analysis identified 3 primary areas for further enhancing the app’s UIs (Multimedia Appendix 7). First, it recommended modifying the width layout of test view components from a fixed value to an adjustable parameter, such as wrap_content. This modification enabled dynamic text expansion, which could prevent component overlap when screen sizes change. Second, it suggested increasing the text size of input items from the current range of 24-44 density-independent pixels to a minimum of 48 density-independent pixels for better readability. Third, it recommended enhancing the contrast colors of the “Today,” “Report,” “Profile,” and “Help” icons to achieve a minimum ratio of 3.00:1 as well as the contrast of unselected time-scrolling texts within the diary reminder interface to at least 4.50:1 with respect to the background.

### BLE Experiment Results

The BLE experiment evaluated data transmission between AIrway (client) and the nRF52840 development board (peripheral; Tables 4 and 5). First, the app’s scan feature accurately detected the board by its device name, “NSA BLE,” along with the unique manufacturing MAC address (D5:D2:5D:62:AF:48). Signal strength, measured in decibel-milliwatts on a logarithmic scale (with values closer to 0 dBm indicating stronger signals [109]), showed that the board was advertising the highest signal value of –19 dBm among the neighboring BLE devices, which confirmed its closest proximity to the app.

**Table 4:** Results of Bluetooth Low Energy scan range.

Scan range (dBm)	Corresponding MAC address
–94	1E:73:99:6D:30:E7
–19	D5:D2:5D:62:AF:4B
–100	57:97:8B:22:DC:5F
–86	5B:7B:F2:E7:2C:3C
–81	6F:AE:90:EE:00:95

**Table 5:** Results of Bluetooth Low Energy data transmission.

Data transmission timestamp	Read characteristics
May 18, 16:33:28	0x0D (equivalent to integer 13)
May 18, 16:33:29	0x0D
May 18, 16:33:30	0x0C (equivalent to integer 12)
May 18, 16:33:31	0x0C
May 18, 16:33:32	0x0B (equivalent to integer 11)

Once connected, the data read characteristic feature was successfully displayed and verified visually. As described in the experiment setup, simulated battery level data were transmitted using random integers and set to decrement by 1 every 2 seconds. The app accurately retrieved the real-time data timestamp along with the corresponding hexadecimal data value (eg, 0x0D representing integer 13, followed by 0x0C representing integer 12 after 2 seconds). This result confirmed the successful integration of the scan, connect, and read characteristic features.

Furthermore, physical distance, connection time, and obstacles were found to impact BLE performance (Table 6). The best performance for stable and continuous data streaming occurred when the devices were adjacent without obstacles. However, after connections exceeded 10 minutes, some packet loss was observed. Additionally, occasional disconnections, particularly when they were farther apart, were likely due to interference from nearby BLE devices. In these cases, the app was able to manually reconnect to the board.

**Table 6:** Results of Bluetooth Low Energy performance test in terms of physical distance, connection time, run time, and physical obstacles.

Task	Physical distance	Scan signal strength (dBm)	Run time (hours:minutes:seconds)	Obstacle
Put the board next to the phone for 5 minutes	0	–26	14:37:35 to 14:42:37	None
Trial 1: put the board next to the phone for 10 minutes	0	–26	16:23:50 to 16:29:19 (self-disconnected)	None
Trial 2: put the board next to the phone for 10 minutes	0	–26	15:40:18 to 15:51:10	None
Put the board next to the phone for 20 minutes	0	–26	14:01:37 to 14:20:07	None
Put the board next to the phone for 30 minutes	0	–26	15:10:17 to 15:42:09	None
Put the board next to the phone for 40 minutes	0	–26	13:13:23 to 13:53:11	None
Put the board and the phone on 2 different laboratory tables for 5 minutes	~2 m apart	–70	14:50:24 to 14:52:49 (self-disconnected)	None
Put the board on the laboratory table and the phone outside the laboratory for 5 minutes	~4 m apart	–91	13:47:24 to 13:48:13 (self-disconnected)	Door
Put the board and the phone next to a microwave (off) for 5 minutes	~0.6 m apart	–58	14:49:44 to 14:55:01	Microwave
Put the board and the phone next to a microwave (on) for 1 minute	~0.6 m apart	–58	15:00:14 to 15:01:31	Microwave

### App Usability Evaluation

A total of 5 app developers participated in the evaluation of AIrway usability. Most technical raters were female, identified as visible minorities, and resided in Canada. They held at least a bachelor degree, worked part-time, and had an average age of 29.0 (SD 5.61) years. The majority were computer science PhD students or postdoctoral fellows with 1-2 years of Android mobile app development experience, and each had developed at least 1 app before (Table 7).

**Table 7:** Demographics and mobile app development experience of technical raters.

Variable and category			Values
**Sex identity, n (%)**			
	Male	2 (40)	
	Female	3 (60)	
	Prefer not to answer	0 (0)	
**Visible minority status, n (%)**			
	Yes	4 (80)	
	No	0 (0)	
	Prefer not to answer	1 (20)	
**Reside location, n (%)**			
	United States	0 (0)	
	Canada	5 (100)	
**Education level, n (%)**			
	High school diploma	0 (0)	
	Apprenticeship or trades certificate or diploma	0 (0)	
	College or CEGEP^a^ degree or diploma, or university degree lower than bachelor	1 (20)	
	Bachelor degree	20 (20)	
	Graduate degree (master or doctorate)	3 (60)	
**Job status, n (%)**			
	Full-time (>30 hours per week) employed	3 (60)	
	Part-time (<30 hours per week) employed	2 (40)	
	Self-employed	0 (0)	
	Unemployed	0 (0)	
**Age (years)**			
	Mean (SD)	29.0 (5.61)	
**Primary role, n (%)**			
	Designing products (eg, UI^b^ designer and interaction designer)	0 (0)	
	Developing software (eg, programmer, developer, and software engineer)	1 (20)	
	Testing software (eg, tester and quality analyst)	0 (0)	
	Managing software development (eg, project manager and IT manager)	1 (20)	
	Computer science PhD student or postdoctoral fellow	2 (40)	
	Other: software and hardware support analyst	1 (20)	
**Experience in mobile development (years), n (%)**			
	1-2	5 (100)	
	2-3	0 (0)	
	3-4	0 (0)	
	More than 5	0 (0)	
**Mobile app development platforms (multiple selected), n (%)**			
	iOS	2 (40)	
	Android	5 (100)	
	Windows	2 (40)	
	BlackBerry	0 (0)	
**Apps developed, n (%)**			
	1	4 (80)	
	2-5	1 (20)	
	More than 5	0 (0)	
**Size of the mobile app development team in the organization, n (%)**			
	1-9 employees	3 (60)	
	10-99 employees	1 (20)	
	100-999 employees	0 (0)	
	1000-9999 employees	0 (0)	
	10,000+ employees	0 (0)	
	Not applicable	1 (20)	

^a^CEGEP: Collège d’Enseignement Général et Professionnel.

^b^UI: user interface.

### uMARS Results

**Figure 5:**
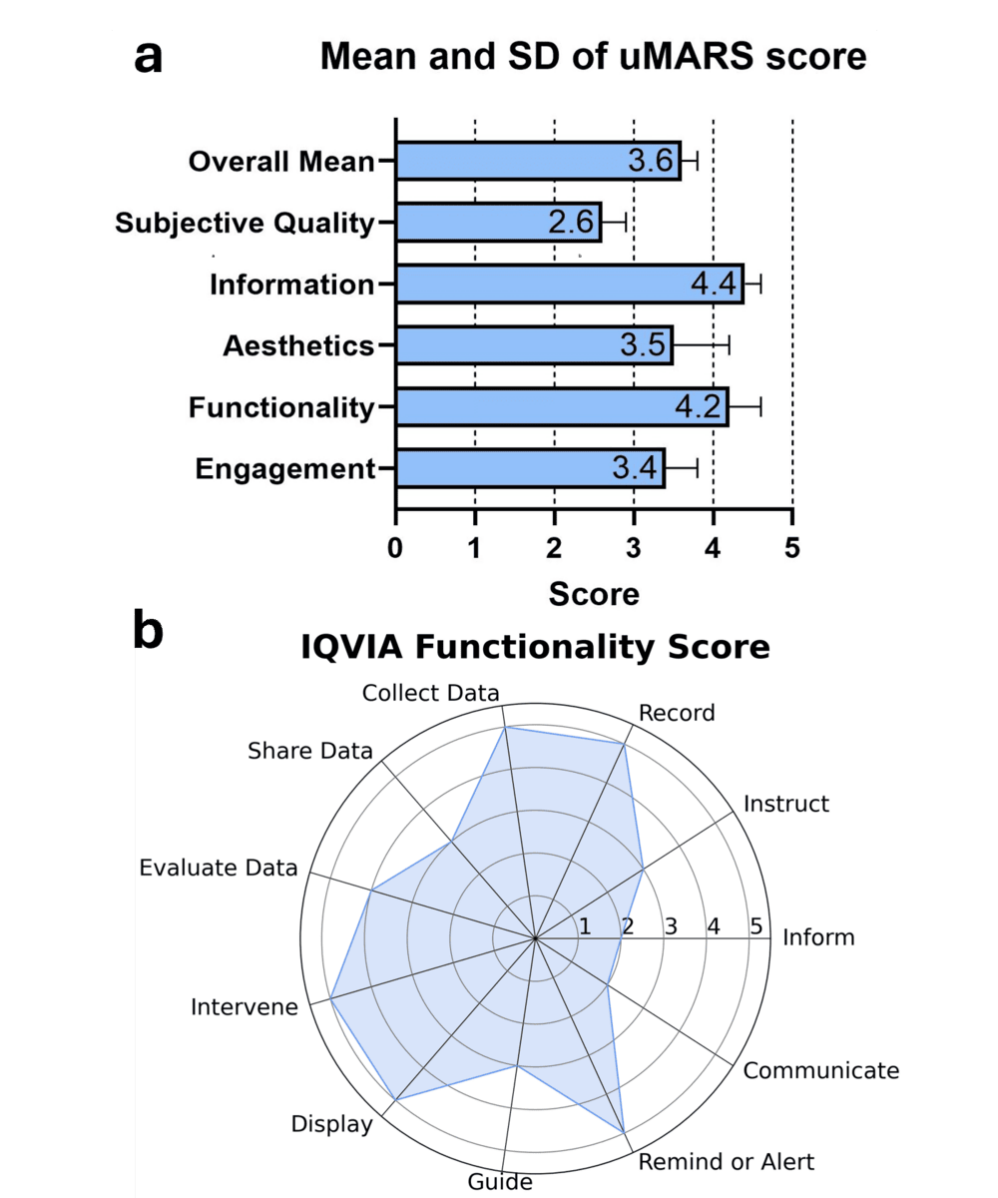
Results of AIrway app usability and functionality. (A) Technical raters’ means and SDs of uMARS and (B) technical raters’ IQVIA functionality scores (n=5).

The overall mean uMARS score for AIrway was 3.6 of 5.0 (SD 0.2), indicating above-average quality (>3.0; Figure 5A). The information quality section received the highest mean score of 4.4 of 5.0 (SD 0.2), which implied that the app’s quality and visual information were highly appealing to users. Similarly, the functionality section also received the highest mean score of 4.2 of 5.0 (SD 0.4), which indicated excellent performance, ease of use, navigation, and gestural design for the app. The aesthetics section received a mean score of 3.5 of 5.0 (SD 0.6), while the engagement section received a mean score of 3.4 of 5.0 (SD 0.4). Both the aesthetics and engagement sections indicated appropriate layout elements and customization aspects. Among all the sections, the subjective quality section received a score of 2.6 of 5.0 (SD 0.3), which indicated a moderate willingness to use the app in the next 12 months and pay for its use.

### IQVIA Results

The IQVIA functionality score ranged from 6 to 11, with a median of 8 (IQR 7-10; Figure 5B). All technical raters confirmed that the app included recording, data collection, intervention, display, and alert functions (5/5, 100%). Most agreed (4/5, 80%) that the user data could be evaluated by others, and over half (3/5, 60%) expressed that the app supported instructing, guiding, and sharing data functions. However, only 1 (20%) rater observed a communication feature that allowed users to interact with others via social networks and provided information in a variety of formats (eg, text, photo, and video).

### Open-Ended Feedback From Technical Raters

Feedback was collected through 5 open-ended questions regarding the app’s engagement, functionality, aesthetics, information quality, and potential improvements. Overall, the technical raters expressed positive impressions of the app and a clear understanding of its purpose. The results are summarized in Multimedia Appendix 8. A key suggestion was to add an option to show and hide passwords in the “Enter a password” and “Retype Your Password” fields on the register interface, which would help users verify that their passwords match.

## Discussion

### Overview

In recent years, mHealth apps targeting asthma monitoring have doubled from 2015 to 2019 [110], and COPD apps increased 6-fold between 2017 and 2023 [111,112]. The SARS-CoV-2 pandemic has notably increased clinicians’ and patients’ familiarity and positive attitudes toward self-monitoring and home-monitoring apps [113]. COVID-19 pandemic restrictions, such as social distancing mandates, shortages of protective equipment, and concerns about disease exposure, had increased the use and adoption of telehealth and remote monitoring technology from 11% to 46% among patients and health care providers [114]. In a study of 162 patients with acute COVID-19 infections, almost 79% of them were reported to engage in the digital platform of remote patient monitoring [114]. Further, to respond to this pandemic, the US Centers for Medicare and Medicaid Services approved over 80 new telehealth services and began reimbursement for telehealth visits since 2020 [114,115].

### Principal Findings and Comparison to Prior Work

In this study, AIrway was designed to support NSA wearable devices for active asthma and COPD self-management purposes. AIrway did not provide diagnostic recommendations or treatment prescriptions to users and was not intended for medical use. Target user characteristics and needs were incorporated into the AIrway app UI design. For instance, the content of AIrway including the privacy policy, FAQ, and action plan was written at a 9th- to 10th-grade reading level that would enhance the comprehensibility and accessibility to general and geriatric users. Meanwhile, about 6.4% of patients with COPD or asthma using bronchodilator medications (eg, β₂-adrenergic agonist bronchodilators) experience hand tremors, which can impair their ability to navigate touchscreen interfaces effectively [116]. The AIrway app has implemented the recommended text sizes (20 scalable pixels for titles, 16 scalable pixels for subtitles, and 14 scalable pixels for buttons and body) and constraint layout to enhance accessibility and adaptability across various screen sizes, following Android Material Design Guidelines. However, these measures were limited, as the app did not include a dynamic font sizing feature to adapt to individual user needs.

BLE tests with the nRF52840 development board demonstrated reliable real-time data exchange and accurate decoding of hexadecimal values. These results support future integration with NSA wearables using nRF5 series microcontrollers. Meanwhile, performance tests revealed that distance, connection time, and obstacles affect BLE stability. Future work will prioritize optimizing critical parameters like the maximum transmission unit and transmission frequency while implementing auto-reconnection functionality to minimize packet loss.

In the usability evaluation, AIrway scored 3.6 of 5.0 on the uMARS and a median IQVIA functionality score of 8 of 11 (IQR 7-10). These results were compared favorably with commercial mHealth apps for respiratory, cardiac, and sleep disorders, which typically report scores ranging from 3.0 to 4.2 on the Mobile App Rating Scale and 6 to 10 on IQVIA [101,116-118]. However, the lower uMARS subjective quality score (2.6/5.0) indicated areas for enhancement. Technical raters valued AIrway’s utility for condition management but showed limited interest in long-term use or payment. Future work will prioritize clinical validation studies co-designed with people living with asthma or COPD and their clinicians, using appropriately powered trials to assess safety and demonstrate meaningful clinical benefits before broader deployment.

### Limitations and Future Directions

The usability study was also limited by a small sample size (n=5) compared to the 15-40 participants typically included in similar research [119-121]. This sampling limitation was attributed to recruitment challenges through university mailing lists and paid research groups on Facebook, which did not effectively reach the intended audience. In addition, the usability evaluation relied on subjective uMARS and IQVIA ratings, which are prone to perception variability and bias. A more comprehensive assessment could incorporate objective use metrics such as system interaction patterns (eg, login frequency and session duration), error occurrence rates, feature use statistics, and configuration preferences to provide a more accurate view of UXs and app performance.

At present, AIrway is Android-specific; thus, future development is needed for cross-platform frameworks like Flutter to improve accessibility and equity by extending availability to a broader user base. Our further accessibility review of the UIs recommended adjusting layout widths to accommodate dynamic text and increasing text sizes to a minimum of 48 density-independent pixels. Future studies should also include patients with asthma and COPD as well as their attending clinicians and caretakers to collect direct feedback from end users, ensuring that AIrway addresses the specific needs and preferences of its target populations.

### Conclusions

This AIrway app followed mHealth best practices to ensure accessibility and seamless wireless integration with wearables. Future work will include cross-platform support and assess usability with patients to validate its clinical effectiveness.

## Appendix

Multimedia Appendix 1 App interfaces.

Multimedia Appendix 2 Privacy policy for the app.

Multimedia Appendix 3 Readability metrics and definition.

Multimedia Appendix 4 Summary of zone calculation and action plan in AIrway.

Multimedia Appendix 5 User Version of the Mobile Application Rating Scale questionnaire.

Multimedia Appendix 6 IQVIA questionnaire.

Multimedia Appendix 7 Accessibility analysis on AIrway app interfaces.

Multimedia Appendix 8 Open-ended question responses of technical raters.

## Abbreviations

ACQ: Asthma Control Questionnaire
AQHI: Air Quality Health Index
BLE: Bluetooth Low Energy
COPD: chronic obstructive pulmonary disease
FAQ: frequently asked question
GINA: Global Initiative for Asthma
HIPAA: Health Insurance Portability and Accountability Act
mHealth: mobile health
NSA: neck surface accelerometer
PM: particulate matter
UI: user interface
uMARS: User Version of the Mobile Application Rating Scale
UX: user experience

## Data Availability

The datasets generated or analyzed during this study are available from the corresponding author on reasonable request.
