# Supplementary material for: An mHealth App and System Architecture for Respiratory Disease Management: Design Principles, Tool Development, and Pilot Usability Study
Source: JMIR Form Res. 2025 Oct 29;9:e73584. doi: 10.2196/73584 (PMC12612645; doi:10.2196/73584)
Supplement: Multimedia Appendix 1 [file formative_v9i1e73584_app1.docx]

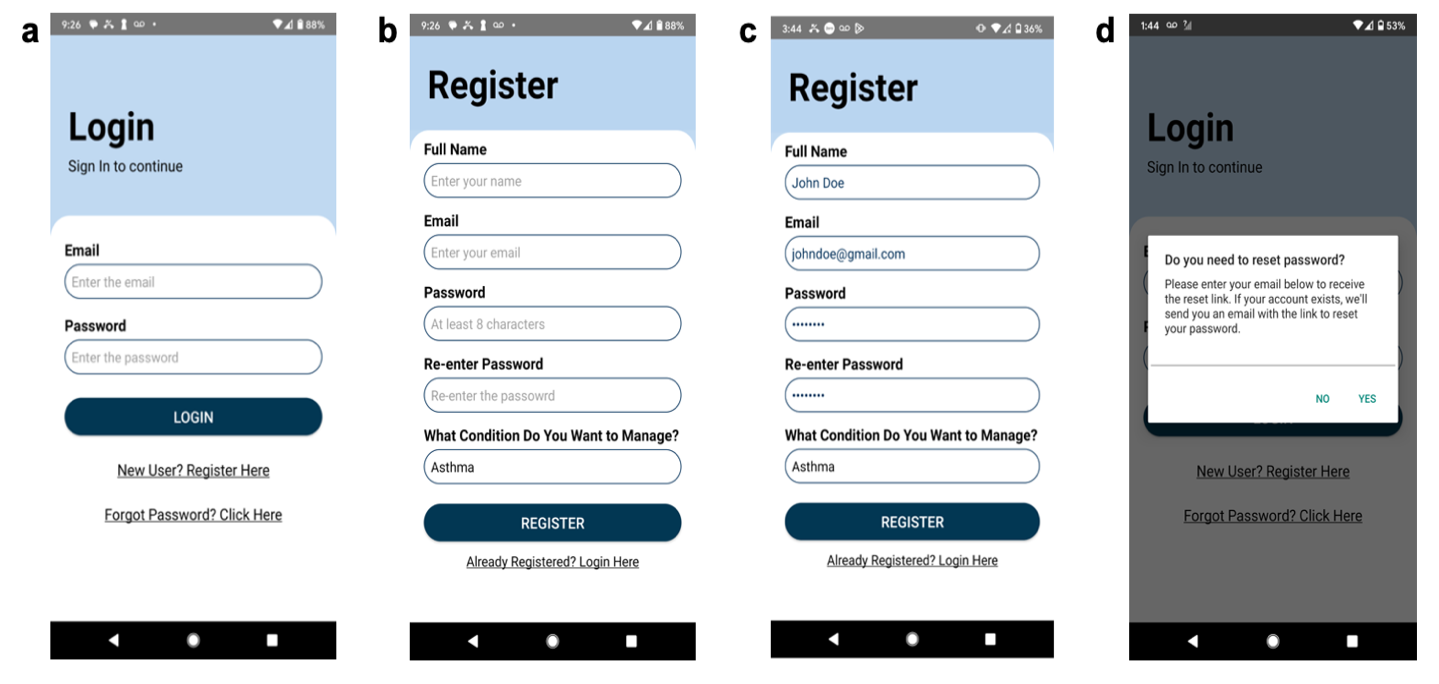


a) Login interface b) Register interface c) Register interface with filled information d) Forgot password prompt.


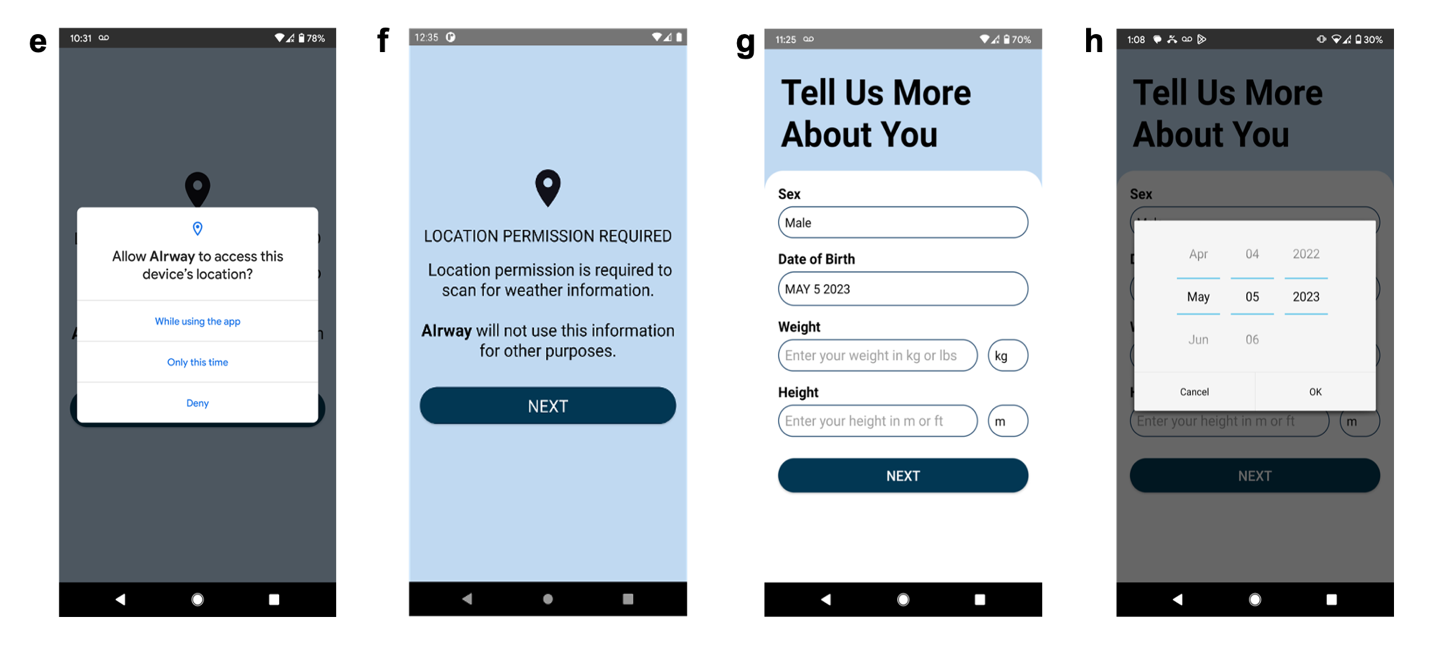


e) Location permission prompt f) Location permission interface g) Account information interface h) DataPicker selection


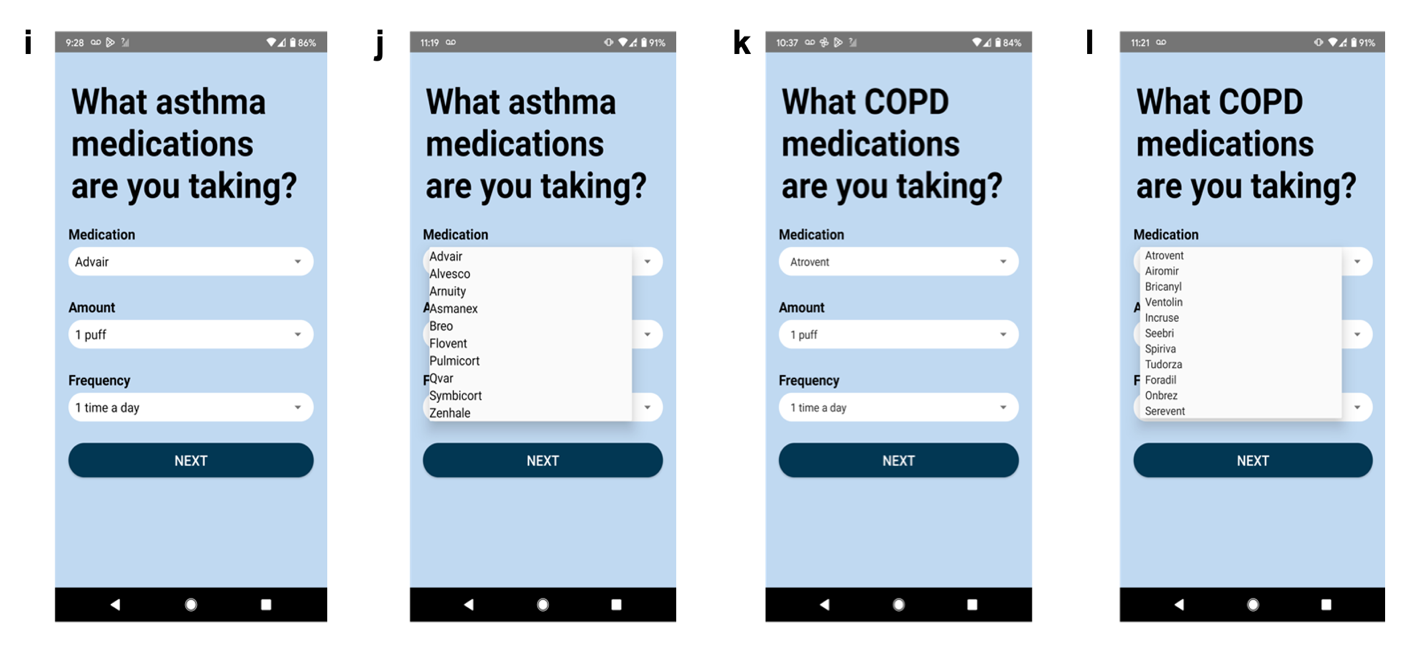


i) Asthma medication interface j) Asthma medication drop-down menu example k) COPD medication interface l) COPD medication drop-down menu example.


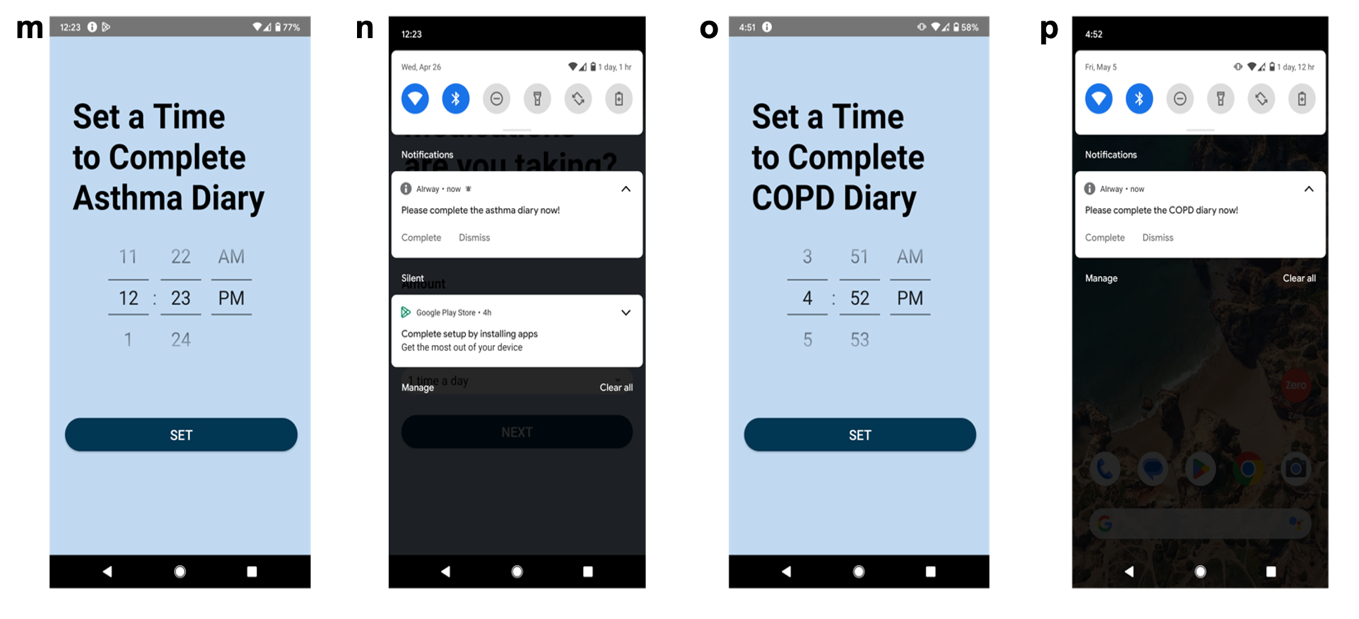


m) Asthma time setting interface n) Asthma diary notification o) COPD time setting interface p) COPD diary notification.
