# Supplementary material for: An mHealth App and System Architecture for Respiratory Disease Management: Design Principles, Tool Development, and Pilot Usability Study
Source: JMIR Form Res. 2025 Oct 29;9:e73584. doi: 10.2196/73584 (PMC12612645; doi:10.2196/73584)
Supplement: Multimedia Appendix 2 [file formative_v9i1e73584_app2.docx]

##

To create a user account in the app, we require some information about you. The information is used to personalize the user experience when using the app. For the purpose of this study, you will enter fictitious data.

The following items will be collected and displayed on the Profile page and will only be visible to you (fictitious data):

- Name
- Email address
- Date of Birth
- Sex
- Weight
- Height
- Medication (usage, dose, frequency)

After logging in, you will also be asked to enable location permission for weather information. The geolocation of the device will be used to display the proper weather information. The following weather information will be displayed on the Today page:

- City name
- Temperature
- Humidity
- AQHI

The following items will be collected and displayed on the Report page

- Your predicted airway conditions
- Your asthma diary response
- Your COPD diary response

Google Firebase services:

Our app uses Google services in order to provide you the cloud functions, such as authentication and data storage. During the use of Google cloud, the following data is collected by Google, extracted from the Google cloud service: <https://firebase.google.com/support/privacy/>

| Firebase Service | Firebase Authentication |
| --- | --- |
| Data required | Passwords, emails, user agents, IP address |
| How is it used to provide the service | Firebase Authentication uses the data to enable end-user authentication and facilitate end-user account management. It also uses user-agent strings and IP addresses to provide added security and prevent abuse during sign-up and authentication. |
| Retention | Firebase Authentication keeps logged IP addresses for a few weeks. It retains other authentication information until the Firebase customer initiates deletion of the associated user, after which data is removed from live and backup systems within 180 days. |

| Firebase Service | Firebase App Distribution |
| --- | --- |
| Data required | Firebase installation IDs, secure Android IDs, emails |
| How is it used to provide the service | Firebase App Distribution uses the data to distribute app builds to testers, monitor tester activity, and associate data with tester devices. |
| Retention | Firebase App Distribution retains user information until the Firebase customer requests its deletion, after which data is removed from live and backup systems within 180 days. |

Note that Google will collect and retain your IP address, among other user information, until the deletion of the account, which is a common practice for using third-party cloud services. As such, we will export all collected app data from the cloud and request the deletion of the account at the end of the study. Once the account is closed, your information will be deleted from the Google backup system within 180 days.

How do we use the information?

We do not use any of your personal information for any reason other than to provide you with the services through the app, including:

1. Registering your user account and allow you to manage the user profile
2. Providing the prediction results
3. Performing statistical analysis about the usability of the app

Who will have access to the information?

All information collected during this study will only be accessible to the researchers at the Voice and Upper Airway Research Lab, McGill University, Canada.

Where do we store the information?

We will temporarily store the information in the Google Firebase Firestore cloud database during the study. After the study, we will export the data and request the deletion of the cloud. The data will then be completely anonymized (destroyed key) and stored safely on password-protected computers to which only the research team has access.

How long do we store the information?

Data will be collected electronically and stored for 7 years following the completion of the study. After this time, all digital files will be destroyed.

How can I revoke my consent or have my information deleted?

To revoke consent and/or have information deleted, please contact support at voicelab.mcgill@gmail.com
