# Supplementary material for: An mHealth App and System Architecture for Respiratory Disease Management: Design Principles, Tool Development, and Pilot Usability Study
Source: JMIR Form Res. 2025 Oct 29;9:e73584. doi: 10.2196/73584 (PMC12612645; doi:10.2196/73584)
Supplement: Multimedia Appendix 3 [file formative_v9i1e73584_app3.docx]

| **Readability Metrics[71]** | **Interpretation** |
| --- | --- |
| Flesch–Kincaid Grade level | The Flesch-Kincaid grade level is the most widely used measures of readability. It is used by the United States military to evaluate the readability of their manuals. It is equivalent to the US grade level of education. |
| Flesch Reading Ease Score | A score between 1 and 100, with 100 being the highest readability score. Scoring between 70 to 80 is equivalent to school grade level 8, which means text should be fairly easy for the average adult to read. Both Flesch use total words and sentences. |
| Gunning Fog Index | Generates a grade level between 0 and 20. It estimates the education level required to understand the text. A Gunning Fog score of 6 is easily readable for sixth graders. Texts aimed at the public should aim for a grade level of around 8. Texts above 17 are for the graduate level. GF uses total words and sentences and helps reduces complexity and help researchers write papers. |
| Coleman Liau Index | Instead of syllables per word and sentence lengths, the Coleman Liau Index relies on characters and uses computerized assessments to understand characters more easily and accurately. Mostly used in school. A score of 6 is 6th grade in the US schooling system. If the writing texts are for the public, aim for a grade level of around 8-10. |
| ARI (Automated Readability Index) | The U.S. grade level required to read a piece of text.  In some ways, it is similar to other formulas. Its difference is rather than counting syllables, it counts characters. The more characters, the harder the word. |
| SMOG Index | Measures how many years of education the average person needs to have to understand a text.  It is best for texts of 30 sentences or more. |
