# Supplementary material for: An mHealth App and System Architecture for Respiratory Disease Management: Design Principles, Tool Development, and Pilot Usability Study
Source: JMIR Form Res. 2025 Oct 29;9:e73584. doi: 10.2196/73584 (PMC12612645; doi:10.2196/73584)
Supplement: Multimedia Appendix 4 [file formative_v9i1e73584_app4.docx]

Summary of asthma zone calculation and action plan in *AIrway*.

| **Zone** | **Thresholds** | **Asthma action plan** |
| --- | --- | --- |
| **Red** | “Uncontrolled”   - Three times or more a week with daytime symptoms and nocturnal symptoms OR - One per week with exacerbation OR - ACQ score > 1.5 OR - Temperature < -12.2°C or > 49 °C OR - Humidity < 30% or > 75 % OR - AQHI > 7 | - A message to user: “You are in the Red Zone. This indicates an increased risk of experiencing an asthma attack. If you believe this prediction is accurate, you should proceed to the nearest hospital for immediate medical attention. Failure to seek medical assistance within the next 15 minutes may escalate the likelihood of a significant asthma attack.” |
| **Yellow** | “Partly Controlled”   - More than twice a week with daytime symptoms OR - Any nocturnal symptoms OR - One per year with exacerbation OR - ACQ score between 0.75 -1.5 | - A message to user: “You are in the Yellow Zone. If you believe this prediction is accurate, you should incorporate prescribed medications into your routine as directed by your healthcare provider. Please pay attention to the temperature, humidity, and pollen levels in your area. If you do not return to the Green Zone after an hour, it is advisable to contact your doctor before considering oral steroids.” |
| **Green** | “Well Controlled”   - No (less than twice a week) daytime symptoms OR - No nocturnal symptoms, exacerbations OR - ACQ score <= 0.75 | - A message to user: “You are in the Green Zone. It's important to maintain this status. If you believe this prediction is accurate, you should continue taking your medication as prescribed, even if you are not experiencing any symptoms. Additionally, be proactive in avoiding triggers that may worsen your condition. Consistent adherence to your treatment plan and preventative measures will contribute to maintaining your condition in a well-controlled state.” |

Summary of COPD zone calculation and action plan in *AIrway*.

| **Zone** | **Thresholds** | **CTS action plan** |
| --- | --- | --- |
| **Red** | - Symptoms are not better after taking your fare-up medicine for 48 hours OR - Experiencing very short of breath, nervous, confused and/or drowsy, and/ or have a chest pain OR - CAT > 20 OR - BCSS <5 OR - NRS <5 OR - Temperature < 0 °C or > 32 °C OR - Humidity < 10% and > 70% OR - AQHI > 7 | - A message to the user: “You are in the Red Zone. This indicates an increased risk of a severe COPD exacerbation. If you believe this prediction is accurate, you should proceed to the nearest hospital for immediate medical attention. Failure to seek medical assistance within the next 15 minutes may escalate the likelihood of a significant COPD exacerbation.” |
| **Yellow** | - Changes in sputum for at least 2 days OR - More shortness of breath than usual for at least 2 days OR - CAT score between 10-20 OR - BCSS <5 OR - NRS <5 | - A message to the user: “You are in the Yellow Zone. If you believe this prediction is accurate, you should incorporate prescribed medications or COPD flare-up medications into your routine as directed by your healthcare provider. Please pay attention to the temperature, humidity, and pollen levels in your area. If your doctor has provided specific breathing and relaxation methods, now is the time to implement them.” |
| **Green** | - No color change in sputum OR - CAT < 10 OR - BCSS <5 OR - NRS <5 | - A message to the user: “You are in the Green Zone. It's important to maintain this status. If you believe this prediction is accurate, you should continue taking your prescribed daily puffers to support your respiratory health. Additionally, be proactive in avoiding triggers that may worsen your condition.   Consistent adherence to your treatment plan and preventative measures will contribute to maintaining your condition in a well-controlled state.” |
