# Supplementary material for: An mHealth App and System Architecture for Respiratory Disease Management: Design Principles, Tool Development, and Pilot Usability Study
Source: JMIR Form Res. 2025 Oct 29;9:e73584. doi: 10.2196/73584 (PMC12612645; doi:10.2196/73584)
Supplement: Multimedia Appendix 5 [file formative_v9i1e73584_app5.docx]

**SECTION A: Engagement**

**1. Is the app fun/entertaining to use?**

1. Dull, not fun or entertaining at all
2. Mostly boring
3. OK, fun enough to entertain user for a brief time (< 5 minutes)
4. Moderately fun and entertaining, would entertain user for some time (5-10 minutes total)
5. Highly entertaining and fun, would stimulate repeat use

**2. Is the app interesting to use?**

- 1. Not interesting at all
  2. Mostly uninteresting
  3. OK, neither interesting nor uninteresting; would engage the user for a brief time (< 5 minutes)
  4. Moderately interesting; would engage the user for some time (5-10 minutes total)
  5. Very interesting, would engage the user in repeat use

**3. What do you think of the app content?**

- 1. Completely inappropriate, unclear or confusing
  2. Mostly inappropriate, unclear or confusing
  3. Acceptable but not specifically designed for the target audience. May be inappropriate, unclear or confusing at times
  4. Designed for the target audience, with minor issues
  5. Designed specifically for the target audience, no issues were found

**4. Do you want to provide more details about app engagement?**

_________________________

**SECTION B: Functionality**

**5. For the home screen, how accurately is the information displayed (e.g., weather, information, location and time)?**

- 1. The display is insufficient/inaccurate (e.g., crashes/broken features, etc.)
  2. The display works, but lagging or contains major technical problems
  3. The function works overall. Some technical problems need fixing/slow at times
  4. Mostly functional with minor/negligible problems
  5. Perfect display. No technical bugs found

**6. For the home screen, are the buttons and menu fast and responsive?**

- 1. The buttons/menu is broken and there is no/insufficient/inaccurate response (e.g., crashes/broken features, etc.)
  2. The buttons/menu works, but lagging or contains major technical problems
  3. The buttons/menu works overall. Some technical problems need fixing/slow at times
  4. Mostly functional with minor/negligible problems
  5. Perfect response. No technical bugs found

**7. Is the app easy to use? Are the menus and instructions clear? Are the labels and icons helpful?**

- 1. No/limited instructions; menu labels/icons are confusing; complicated
  2. Takes a lot of time or effort
  3. Takes some time or effort
  4. Easy to learn (or has clear instructions)
  5. Able to use the app immediately; intuitive; simple (no instructions needed)

**8. Is login/ register a user account uninterrupted?**

- 1. The navigation is difficult
  2. Usable after a lot of time/effort
  3. Usable after some time/effort
  4. Easy to use
  5. Perfectly logical, easy, clear flow throughout

**9. Are interactions (taps/scrolls) consistent and intuitive across all screens?**

- 1. Completely inconsistent/confusing
  2. Often inconsistent/confusing
  3. OK with some inconsistencies/confusing elements
  4. Mostly consistent/intuitive with negligible problems
  5. Perfectly consistent and intuitive

**10. Do you want to provide more details about app functionality?**

_________________________

**SECTION C: Aesthetics**

**11. Is the arrangement and size of buttons/icons on the screen appropriate?**

1. Very bad design, cluttered, some options impossible to select, locate, see or read
2. Bad design, random, unclear, some options difficult to select, locate, see or read
3. Satisfactory, few problems with selecting, locating, seeing or reading items
4. Mostly clear, able to select, locate, see or read items
5. Professional, simple, clear, orderly, logically organized

**12. How high is the quality of graphics used for the content?**

1. Graphics appear amateur, very poor visual design – completely stylistically inconsistent
2. Low-quality graphics or low-quality visual design – stylistically inconsistent
3. Moderate quality graphics and visual design – generally consistent in style
4. High-quality graphics and visual design – mostly stylistically consistent
5. Very high-quality graphics and visual design – stylistically consistent throughout

**13. How does the app look on the screen?**

1. Ugly, unpleasant to look at, poorly designed, clashing, mismatched colors
2. Bad – poorly designed, bad use of color, visually boring
3. OK – average, neither pleasant, nor unpleasant
4. Pleasant – seamless graphics – consistent and professionally designed
5. Beautiful – very attractive, memorable, stand out; use of color enhances app features/menus

**14. Do you want to provide more details about app aesthetics?**

_________________________

**SECTION D: Information**

**15. Is the weather data relevant to the app?**

1. Irrelevant**,** inappropriate, incoherent or incorrect
2. Poor. Barely relevant, appropriate, coherent, or may be incorrect
3. Moderately relevant, appropriate, coherent**,** or appears correct
4. Relevant**,** appropriate, coherent, correct
5. Highly relevant, appropriate, coherent, or correct

**16. Is the clinical diary relevant to the app?**

1. Irrelevant**,** inappropriate, incoherent or incorrect
2. Poor. Barely relevant, appropriate, coherent, or may be incorrect
3. Moderately relevant, appropriate, coherent**,** or appears correct
4. Relevant**,** appropriate, coherent, correct
5. Highly relevant, appropriate, coherent, or correct

**17. Does the help page provide clear, logical, and correct information?**

1. Completely unclear, confusing, wrong
2. Mostly unclear, confusing, or wrong
3. OK but often unclear, confusing, or wrong
4. Mostly clear, logical, or correct with negligible issues
5. Perfectly clear, logical, or correct

**18. Does the error messaging provide clear, logical, and correct information?**

1. Completely unclear, confusing, wrong
2. Mostly unclear, confusing, or wrong
3. OK but often unclear, confusing, or wrong
4. Mostly clear, logical, or correct with negligible issues
5. Perfectly clear, logical, or correct

**19. Do you want to provide more details about app information?**

_________________________

**SECTION E: App subjective quality**

**20. Do you think the app can be used for self-management purpose (e.g., used by patients daily for monitoring their health conditions)?**

1. The app has no chance of achieving it
2. The app has very little chance of achieving it
3. This app may be able to achieve it
4. This app has high chance of achieving it

**21. Would you pay for this app?**

- - 1. No
    2. Maybe
    3. Yes

**22. What is your overall star rating of the app?**

1. 1 star
2. 2 stars
3. 3 stars
4. 4 stars
5. 5 stars

**23. Would you make changes or add anything to this app? If yes, please describe it below.**

_____________________
