# Supplementary material for: An mHealth App and System Architecture for Respiratory Disease Management: Design Principles, Tool Development, and Pilot Usability Study
Source: JMIR Form Res. 2025 Oct 29;9:e73584. doi: 10.2196/73584 (PMC12612645; doi:10.2196/73584)
Supplement: Multimedia Appendix 6 [file formative_v9i1e73584_app6.docx]

Please answer the following questions. If you believe the function is presented in the app, please select “Yes”. If not, please select “No”.

| Rating description | User Response |
| --- | --- |
| 1. Provide information in a variety of formats (e.g., text, photo, video) |  |
| 2. Provide instructions to the user (e.g., app user guide) |  |
| 3. Capture user-entered data (e.g., diary response) |  |
| 4. Able to enter and store health data on the individual’s phone |  |
| 5. Able to transmit health data (e.g., export, upload, email data) |  |
| 6. Able to evaluate the entered data by patient and provider, provider and administrator, or patient and caregiver |  |
| 7. Able to send alerts based on the data collected or propose behavioral intervention or changes (e.g., self-management action) |  |
| 8. Graphically display user-entered data/ output user-entered data |  |
| 9. Provide guidance based on user-entered information, and may further offer a diagnosis, or recommend a consultation with a physician/ a course of treatment |  |
| 10. Provide reminders to the user |  |
| 11. Provide communication between health care providers, patients, consumers, and caregivers and/ or provide links to social networks |  |
