# Supplementary material for: An mHealth App and System Architecture for Respiratory Disease Management: Design Principles, Tool Development, and Pilot Usability Study
Source: JMIR Form Res. 2025 Oct 29;9:e73584. doi: 10.2196/73584 (PMC12612645; doi:10.2196/73584)
Supplement: Multimedia Appendix 7 [file formative_v9i1e73584_app7.docx]

| **User Interfaces** | **Accessibility Scanner Results** |
| --- | --- |
| Login | The “*New User? Register*” and “*Forgot Password? Click Here*” text views currently have fixed widths and scalable text. It's recommended to adjust the layout settings to allow for text expansion, such as using $wrap\_content$.  The input item height for “*Email*” and “*Password*” is currently 44dp. Consider increasing the height of this target to 48dp or larger. |
| Forgot Password Popup | The input item height for “*Reset Password*” is currently 40dp. Consider increasing the height of this target to 48dp or larger. |
| Register | The input item height for “*Full Name*,” “*Email*,” and the selection input for “*What Condition Do You Want to Manage*” is currently 44dp. Consider increasing the height of these touch targets to 48dp or larger. |
| Tell Us More About You | The selection item for the user’s “*Sex*” and units for “*kg*,” “*lbs*,” “*m*,” and “*ft*” input is currently set at 24dp. Consider increasing the height of this target to 48dp or larger.  The input item height for “*Weight*” and “*Height*” is currently 40dp. Consider increasing the height of these targets to 48dp or larger. |
| Medication | The selection item for “*Medication*,” “*Amount*,” and “*Frequency*” input is currently 24dp. Consider increasing the height of this target to 48dp or larger. |
| Diary Reminder | Consider increasing the current contrast ratio (3.80:1) to a higher ratio (e.g., 4.50:1) between the unselected time-scrolling texts (light grey color) and the app background (blue color). |
| Clinical Diary Questionnaires | The selection item for the questionnaire input is currently 32dp. Consider increasing the height of this target to 48dp or larger. |
| Today Page | Consider increasing the current contrast ratio (2.34:1) to a higher ratio (e.g., 3.00:1) between the “Today” icon (green color) and the selection menu background (light grey color).  The “*System Monitoring*” and “*Recommended Action*” text views currently have fixed widths and scalable text. It's recommended to adjust the layout settings to allow for text expansion, such as using $wrap\_content$. |
| Report Page | Consider increasing the current contrast ratio (2.34:1) to a higher ratio (e.g., 3.00:1) between the “*Report*” icon (green color) and the selection menu background (light grey color). |
| Profile Page | Consider increasing the current contrast ratio (2.34:1) to a higher ratio (e.g., 3.00:1) between the “*Profile*” icon (green color) and the selection menu background (light grey color).  The explainable view list has fixed widths and scalable text. It's recommended to adjust the layout settings to allow for text expansion, such as using $wrap\_content$. |
| Help Page | Consider increasing the current contrast ratio (2.34:1) to a higher ratio (e.g., 3.00:1) between the “*Help*” icon (green color) and the selection menu background (light grey color).  The explainable view list has fixed widths and scalable text. It's recommended to adjust the layout settings to allow for text expansion, such as using $wrap\_content$. |
