# Supplementary material for: An mHealth App and System Architecture for Respiratory Disease Management: Design Principles, Tool Development, and Pilot Usability Study
Source: JMIR Form Res. 2025 Oct 29;9:e73584. doi: 10.2196/73584 (PMC12612645; doi:10.2196/73584)
Supplement: Multimedia Appendix 8 [file formative_v9i1e73584_app8.docx]

| **Do you want to provide more details about app engagement?** |
| --- |
| **Feedback #1:** Most functionalities are correctly implemented |
| **Feedback #2:** It was a nice app meets the users’ needs. There might still need to be a few UI tweaks like spacing and font sizes. Also, the email input in the forgot password popup is not very intuitive, it would be nice to have an input box similar to the email input box on the login page with proper labeling. Also, when the yes button is clicked it would be nice to show an error message asking to input email rather than just closing the app. |
| **Feedback #3:** The first question asks about whether the app was fun/entertaining - I guess I never thought about the app as having to be that way. So my selection of "mostly boring" is not a bad thing, i.e. doesn't give a negative connotation to the app. For me a "fun" app would be gamified or with more interactive elements, but I would not expect that from a medical related app anyway.  In terms of engagement, the notification helps to stay engaged, although I wish it would automatically dismiss once I clicked "Complete" and was redirected to the diary entry. |
| **Feedback #4:** The font size at the top of the app was quite large and it made viewing the temperature information difficult. I didn’t know I can click on the information icon and additional information popped up. I wouldn’t have done this if I did not receive the instructions. The interface of the app doesn't look very enticing to the user, no color was used other than the report page. |
| **Feedback #5:** Very effective and informative. Could possibly benefit from and slightly more varied colour pallet. Today screen could be benefit from minor images or icons. |
| **Do you want to provide more details about app functionality?** |
| **Feedback #6:** I think there should be a nice way to distinguish between the page titles and content. Also, it's need more spacing in a few places for example my link to call emergency contact was kind of cut off and there was no space between temperature content and humidity content. |
| **Feedback #7:** Would be great to have a "View your password" especially when creating a new account to make sure my spellings are correct. I spent a couple of times trying to set the passwords because both of them were not matching.  Labels on the bottom navigation icons would be helpful on hover/click (e.g., it only said "Today" some time if I remember correctly, but not when I initially opened the app). It took me a minute to realize the second icon is a report, I thought it was a diary entry/notebook icon.  The reset password is a bit unintuitive - I clicked yes without typing in an email/password thinking that it would then take me to the next page where I could reset my password (that made the app crash though). So maybe having a textbox instead of an underline would make it clear that the user is expected to put in the email there. Overall, pretty simple app with no complex navigation required! |
| **Feedback #8:** I think the interactions across different screens can be difficult among older adults that are not used to using mobile apps and phones. The font was relatively large, it would be better if there was an option to decrease the font size. Login page was smooth and easy to use. |
| **Feedback #9:** Fairly smooth. Menu buttons are responsive and point the appropriate info. |
| **Do you want to provide more details about app aesthetics?** |
| **Feedback #10:** The display is a bit wordy, more graphical icons can be used in placed of text |
| **Feedback #11:** For the things like temperature, humidity, PM10, PM2.5 they seem all clustered together for some reason. I feel icons or smaller text might actually do the trick. And for the tab bar on the bottom if there could be more spacing between the texts and the icons on the active screen it would look nicer. |
| **Feedback #12:** Forgot to mention previously that it wasn't clear what was clickable and what isn't - maybe highlighting that in some way would help.  Also, I prefer if previous toggles are closed when new toggles are opened (e.g., in the FAQ and Privacy Policy), but that is my personal preference, and I'm nit-picking here. |
| **Feedback #13:** The quality of the graphics was not too high; the font was clear though and easy to understand. I think the app can look better with smaller font size and more colour on the first screen. |
| **Feedback #14:** Graphics seem alright, but layout needs to be optimized. Some overlap of text especially on Today screen. |
| **Do you want to provide more details about app information?** |
| **Feedback #15:** I feel on the help page "support" should a bigger font since it is the main title. |
| **Feedback #16:** I find the weather-related information somewhat redundant because an Android phone user could just have a weather app for that information. Probably if this provided more illness-specific details, e.g., percentage of pollen in the air, recommendation for how to protect oneself in the current weather, etc. that might be very useful.  The error for resetting password was unclear (the App crashed instead of redirecting me to type out the associated email). |
| **Feedback #17:** The weather app is relevant for asthma. The diary page was easy to use and the prompt worked well to remind me to complete it. The FAQ and error messaging was clear, no issues. |
| **Feedback #18:** App seems very functional, minor layout and format adjustments should seem to be only issues so far. |
| **Would you make changes or add anything to this app? If yes, please describe it below.** |
| **Feedback #19:** Nice app overall. Love the concept! |
| **Feedback #20:** I guess a feature on how often to fill in the diary entry would help (e.g., how many times a day daily), based on the needs of the user. |
| **Feedback #21:** I would add the pollen levels and air quality to the app so patients can understand more about the weather prior to stepping outside. I think the interface can be more esthetically pleasing with more colours and font changes. If the app was recommended by a healthcare provider, I think some patients will be open to trying it. I don't think people would use it if they had to pay for it. |
| **Feedback #22:** Add a few more graphics to engage the user more and simple optimization; format and layout issues, mainly on Today screen, some texts got cut out. Would be good to have a history of symptoms. |
